# Stiffness Compensation in Variable Displacement Mechanisms of Swash Plate Axial Piston Pumps Utilizing Piezoelectric Actuators

**DOI:** 10.3390/ma18030520

**Published:** 2025-01-23

**Authors:** Guangcheng Zhang, Mengxiang Ma, Yueh-Jaw Lin

**Affiliations:** 1 School of Mechanical Engineering, University of Shanghai for Science and Technology, Shanghai 200093, China; g.c.zhang@usst.edu.cn (G.Z.); 222171430@st.usst.edu.cn (M.M.); 2 College of Engineering and Engineering Technology, Northern Illinois University, Dekalb, IL 60115, USA

**Keywords:** piezoelectric actuator, ball screw, stiffness compensation, Bouc–Wen model, variable displacement mechanisms

## Abstract

Swash plate axial piston pumps play an important role in hydraulic systems due to their superior performance and compact design. As the controlled object of the valve-controlled hydraulic cylinder, the swash plate is affected by the complex fluid dynamics effect and the mechanical structure, which is prone to vibration, during the working process, thereby adversely affecting the dynamic performance of the system. In this paper, an electronically controlled ball screw type variable displacement mechanism with stiffness compensation is proposed. By introducing piezoelectric ceramic materials into the nut assembly, dynamic stiffness compensation of the system is achieved, which effectively changes the vibration characteristics of the swash plate and thus significantly improves the working stability of the system. Based on this, the stiffness model of a double nut ball screw is established to obtain the relationship between piezoelectric ceramics and the double nut. An asymmetric Bouc–Wen piezoelectric actuator model with nonlinear hysteresis characteristics is also established, and a particle swarm algorithm with improved inertia weights is utilized to identify the parameters of the asymmetric Bouc–Wen model. Finally, a piezoelectric actuator model based on the feedforward inverse model and a PID composite control algorithm is applied to the variable displacement mechanism system for stiffness compensation.

## 1. Introduction

As the heart of the hydraulic system power element, axial piston pumps play an irreplaceable role in the aerospace, engineering machinery, national defense, and military industries owing to their high power density [1], wide variable range [2], and flexible layout [3]. In recent years, with the growing emphasis on developing an environmentally friendly and resource-efficient society, researchers in the field of hydraulics, aiming at energy saving and emission reduction, have been committed to technological innovation and optimal design to reinforce the energy efficiency and reliability of the system, and this direction has become an important research hotspot [4,5]. Swash plate axial piston pumps typically rely on a valve-operated hydraulic cylinder design for variable control. Although this design has demonstrated its technical superiority in numerous application scenarios, the inherent response delay in hydraulic systems and nonlinear nature of the valve-controlled cylinder in the control process limit the system’s response to changes in external conditions [6,7]. At the same time, the pump’s variable displacement mechanism plays a crucial role in enhancing the pump’s flow response. By optimizing the variable displacement mechanism, the flow rate can be regulated faster and more accurately [8]. Therefore, exploring and optimizing the variable regulation mechanism of axial piston pumps to overcome these inherent limitations has become a key task in improving the overall performance and energy efficiency of hydraulic systems.

Scholars have made significant progress in exploring advanced control methods for the variable displacement mechanism of swash plate axial piston pumps. Chu et al. [9] designed a neural network-based adaptive controller to regulate the swash plate angle. The time response of the swash plate angle was analyzed through both simulation and experimental validation. The results confirm that the proposed neural network controller effectively tracks the target model, demonstrating superior tracking performance and excellent dynamic response. Ahn et al. [10] proposed an adaptive backstepping control method for electro-hydraulic actuators, which compensates for nonlinearities and uncertainties by means of an improved algorithm and a special adaptive law, thus improving the robustness of the pump control system. Song et al. [11] proposed a hydraulic pump displacement control method based on a PI algorithm and feedforward compensation, aiming to enhance the dynamic response and tracking accuracy of actuators in construction machinery. Simulation and experimental outcomes demonstrate that the newly developed controller outperforms the conventional PI controller, effectively minimizing actuator oscillation. Yamada et al. [12] designed a model predictive control (MPC)-based displacement control system for axial piston pumps, considering a variety of constraints and introducing an adaptive system and variable control input constraints to improve control performance. Feng et al. [13] proposed a control strategy in view of H∞ control theory to ramp up the response speed and control accuracy of the variable displacement mechanism of the swash plate axial piston pump. The study solved the problems of difficult parameter adjustment, serious hysteresis and large overshooting in the traditional PID control, significantly shortened the regulation time, and significantly improved the system’s performance. Helian et al. [14] devised an adaptive robust pressure control strategy based on an enhanced simplified dynamic model. This approach effectively addresses the challenge of precise pressure tracking in the variable displacement mechanism of axial piston pumps, especially under conditions characterized by dynamic nonlinearity and parameter uncertainty. The effectiveness of the proposed control strategy has been rigorously validated through a series of experiments and simulations conducted under diverse operating conditions. The above algorithms have a joint feature of considering the nonlinearity of the system through the use of nonlinear models. Nevertheless, applying such nonlinear algorithms in engineering practice is still challenging under the current technological constraints.

Some scholars are actively exploring the integration of electro-hydraulic technology into the design of variable displacement mechanisms in pumps. This approach aims to fundamentally transform the traditional valve-controlled variable displacement method in variable displacement pumps, serving as a key strategy to enhance the system’s dynamic response characteristics and overall performance. Gao et al. [15] proposed a new type of variable displacement mechanism—this variable displacement mechanism drove the active gear through a DC servomotor to achieve rapid adjustment of the pump displacement—and achieved good results through simulation analysis. There is backlash in the gearing process, which inevitably has a negative impact on system performance when the pump is started or reversed. On this basis, Song et al. [16] proposed replacing the gearing mechanism with a set of conical worm gear mechanisms to minimize the negative effect of backlash on the system’s performance and also placed the variable displacement mechanism inside the pump casing to reduce the effect of external environment on the pump performance. However, the conical worm gear mechanism also has the problems of lower transmission efficiency and higher friction loss. Ma et al. [17] proposed an advanced axial piston variable pump, which builds on the foundational design of the A4V pump but incorporates a servo valve for direct control of the variable displacement mechanism. This innovative pump design enables precise and timely load sensing in response to output pressure commands issued by the intelligent pump controller. By accurately capturing load dynamics, the system provides critical data essential for real-time monitoring of pump operating status and facilitates proactive troubleshooting and maintenance. Yang et al. [18] designed an open-loop control mechanism driven by a servo motor floating cup pump for variable control, and the precise control of swash plate angle was achieved—the response time from 0° to 18° was only 0.08 s. Meanwhile, certain issues in the design were identified, which provided a robust theoretical foundation for the optimal design of the variable displacement mechanism of the floating cup pump.

The above studies were conducted to improve the dynamic characteristics and performance metrics of swash plate axial piston pumps from the perspectives of control algorithm and structural design, respectively. There are still problems attributed to the lack of stiffness compensation for the variable displacement control mechanism. The stiffness of the traditional valve-controlled cylinder variable displacement mechanism mainly depends on the structural design and material properties, while in the hydraulic system, the working conditions are often complex and constantly changing, which leads to the existence of certain limitations of passive stiffness control. In contrast, active stiffness control is better able to adapt to these changes and can adjust stiffness in real time to ensure that the system is always in the best working condition.

Piezoelectric ceramics as smart materials have found extensive application in the fields of micro-robotics [19] and scanning tunneling microscopes (STMs) [20] owing to their high frequency response, high stiffness, and large mass ratio [21]. Therefore, output force control with fast response can be achieved by piezoelectric ceramics. However, the inherent hysteresis, creep, and other [22] nonlinear characteristics of piezoelectric ceramics pose a challenge for improving their control accuracy. The ball screw mechanism is one of the most widely used drive systems and has the advantages of high speed, large stroke, and high transmission efficiency [23,24]. Through the design of its mechatronics, it replaces the traditional valve-controlled hydraulic-type variable displacement mechanism, which significantly increases control precision and response speed of variable displacement pump. The vibration of the swash plate of an axial piston pump is closely related to the stiffness of the swash plate, and the ball screw directly transmits the alternating load of the swash plate through a mechanical connection. Vibration of the swash plate affects the ball screw through this mechanical connection, leading to a loss of system stability and adversely affecting the performance of the overall system. To improve this situation, piezoelectric ceramics were introduced as a variable stiffness actuator into the nut assembly to enable dynamic stiffness compensation control of the ball screw.

In this paper, a stiffness model of a two-nut ball screw is developed to obtain the relationship between the piezoelectric ceramic and the two nuts. The paper also establishes an asymmetric Bouc–Wen piezoelectric actuator model with nonlinear hysteresis characteristics and uses a particle swarm algorithm with improved inertia weights to identify parameters of the asymmetric Bouc–Wen model. Finally, the piezoelectric actuator model based on feedforward inverse model and PID composite control algorithm is applied to the variable displacement mechanism system for stiffness compensation. The ball screw variable displacement mechanism with piezoelectric ceramic compensation shows significant advantages in the application of swash plate axial piston pumps. Simulation results demonstrate that the stiffness compensation provided by piezoelectric ceramics significantly enhances the stability of the plunger pump swash plate. This improvement contributes substantially to the overall efficiency and reliability of the pump. The system exhibits the capability to maintain stable output flow and pressure across a range of operating conditions, underscoring its considerable potential for industrial applications.

## 2. Composition and Working Principle of Variable Displacement Mechanism

**Figure 1:**
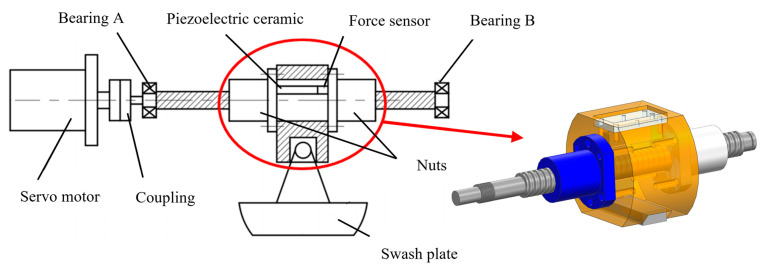
Schematic diagram of variable displacement mechanism system components.

As the core actuator for variable displacement control, the adjustment range of the swash plate angle directly determines the variability of the output displacement of the axial piston pump. This parameter is crucial in achieving diversified pump performance and adapting to the requirements of complex working conditions. Schematic diagram of variable displacement mechanism system components is shown in Figure 1. The mechanism consists of a servo motor, coupling, ball screw, nut assembly, support bearing, fixed bearing and swash plate. The servo motor adjusts the angle of the swash plate by precisely controlling its speed and angle, thus adjusting the output displacement of the pump. The coupling not only transmits torque but also absorbs installation errors and minor deviations during operation. A ball screw converts rotary motion into linear motion, which pushes the swash plate through the nut assembly to achieve the angle change. Support bearings and fixed bearings provide essential support and guidance, effectively reducing friction and wear. To further enhance the dynamic response and stability of the system, piezoelectric ceramic elements have been integrated into the nut assembly. Leveraging their inverse piezoelectric effect, these elements enable real-time adjustment of system stiffness, thereby achieving dynamic compensation. This innovation ensures the efficient operation of the axial piston pump in complex working situations.

## 3. Mathematical Modelling of Variable Displacement Mechanism System

### 3.1. Ball Screw Mechanical Drive System Model

The ball screw system takes the rotary motion of the servo motor as the driving force and transmits torque and speed to one end of the screw through its output shaft. Considering the complexity of the ball screw mechanism and its influence by many factors, the following simplification measures are adopted in constructing the dynamic model: firstly, the damping effect of the coupling is set to a fixed value, ignoring the influence of the speed change and other factors on its dynamic characteristics; secondly, the potential influence of the external conditions, such as the ambient temperature, on system performance has not been considered. For the sake of simplifying the analysis, all the gaps (including the gap between the coupling and the bearing) are uniformly simplified as the relative gap between the ball screw and the nut; finally, to simplify the analysis, all clearances (including clearance between the coupling and the bearing) are unified and simplified as the relative displacement between the ball screw and the nut. These simplification measures help to focus on key parameters and speed up the process of model building and analysis. On this basis, the dynamic equations of the servo motor shaft and ball screw are established:

(1) $$\begin{array}{c} T_{d}=K_{1}\cdot F_{d}=\frac{P_{h}}{2\pi }\cdot \left(F_{m}+F_{x}\right), \end{array}$$

(2) $$\begin{array}{c} J_{1}{\ddot{\theta }}_{0}+C_{0}{\dot{\theta }}_{0}+T_{in}+T_{f}(\pm \mathrm{sgn}\theta_{0})=T_{m}, \end{array}$$

(3) $$J_{2}{\ddot{\theta }}_{1}+C_{1}{\dot{\theta }}_{1}=T_{in}-T_{d}.$$

where $T_{d}$ is the torque of the motor driving the moving parts movement, $K_{1}$ is the conversion factor between rotary and linear motion of the screw, $K_{1}=P_{h}/(2\pi )$, where $P_{h}$ is the ball screw lead, $F_{d}$ represents the driving force exerted by the ball screw on the nut assembly, $F_{m}$ is the inertia force of the nut assembly movement and the force needed to overcome the friction and damping, $F_{x}$ is the force needed for nut assembly displacement change, $J_{1}$ is the equivalent rotational moment of inertia of the motor, $C_{0}$ is the viscous damping coefficient of the coupling, $\theta_{0}$ is the angular displacement of the output end of the motor shaft, $T_{in}$ is the input torque of the ball screw, $T_{f}$ is the rotational friction torque of the ball screw, $T_{m}$ is the driving torque of the servomotor, $J_{2}$ is the rotational moment of inertia of the ball screw, $C_{1}$ is the viscous damping coefficient of the ball screw, and $\theta_{1}$ is the angular displacement of the input end of the ball screw.

Equations (1)–(3) are associated, and Laplace transform is used to convert complex time domain differential equations into frequency domain algebraic equations, thus simplifying the solution process and facilitating system analysis. The Laplace transform under zero initial condition yields:

(4) $$\begin{array}{c} T_{m}=\left(J_{1}\cdot s^{2}+C_{0}\cdot s\right)\theta_{0}+\left(J_{2}\cdot s^{2}+C_{1}\cdot s\right)\theta_{1}+\\ \frac{P_{h}}{2\pi }\cdot \left(F_{m}+F_{x}\right)+T_{f}(\pm \mathrm{sgn}\theta_{0}). \end{array}$$

As shown by Equation (4), incorporating the elastic properties of the coupling and the ball screw itself essentially manifests itself as the coupling of the system into two second-order linear dynamical subsystems. This approach allows a more accurate representation of the system’s behavior, capturing dynamic interactions between its components with greater fidelity.

### 3.2. Stiffness Model

The drive force of a ball screw on a moving part (slider) is expressed by the equivalent damping and stiffness:

(5) $$\begin{array}{c} F_{d}=K_{T}(P_{h}\theta_{0}-x_{0})+C_{T}(P_{h}{\dot{\theta }}_{0}-{\dot{x}}_{0}). \end{array}$$

where $K_{T}$ is the equivalent stiffness of the feed system and $C_{T}$ is the equivalent damping coefficient of the feed system. From the conversion factor $K_{1}$, torsional stiffness $K_{\theta }$, axial stiffness $K_{L}$, the equivalent stiffness is obtained as:

(6) $$\begin{array}{c} \frac{1}{K_{T}}=K_{1}^{2}\frac{1}{K_{\theta }}+\frac{1}{K_{L}}. \end{array}$$

where $K_{1}$ is used as a conversion factor between rotational and linear motion, which ensures that both torsional and axial stiffnesses are in the same unit system, thus accurately calculating the equivalent stiffness.

The ball screw shaft is the primary factor influencing the torsional deformation of the ball screw assembly, with its resistance to such deformation characterized by its torsional stiffness. The following discussion focuses on the torsional stiffness of the screw shaft. In order to simplify calculations, the screw shaft will be modeled equivalently as a solid shaft, and its torsional stiffness will be determined according to Equation (7):

(7) $$\begin{array}{c} K_{\theta }=\frac{GJ_{p}}{B}=\frac{{G\pi d}^{\;4}}{32B}. \end{array}$$

where G is the shear modulus of elasticity, $J_{p}$ is the cross-section moment of inertia, d is the nominal diameter of the screw, B is the distance from one end of the screw to the center of the nut, and the furthest distance from this end of the screw that the nut is in full travel.

The axial stiffness of a ball screw is measured by its ability to resist axial elastic deformation. This characteristic is influenced by a number of factors, mainly the inherent stiffness of the screw itself and the contact stiffness between the balls, screw, and nut. Additionally, other elements—such as the preload applied, the diameter of the ball, the number of balls, and the design of the recirculation mechanism—can also play significant roles in determining the overall axial stiffness. To improve the positioning accuracy and stability of the ball screw and reduce the displacement induced by the tilting disc alternating force, the variability of its stiffness must be considered, and by adding piezoelectric ceramics between the double nuts, the stiffness of the nut assembly can be dynamically adjusted to achieve real-time optimization of the axial stiffness of the ball screw. The axial stiffness of the ball screw is:

(8) $$\begin{array}{c} \frac{1}{K_{L}}=\frac{1}{K_{s}}+\frac{1}{K_{N}}={\left(\frac{\pi {d_{r}}^{2}E}{4B}\right)}^{-1}+\;0.8\times K\;{\left(\frac{F_{pzt}}{0.1C}\right)}^{-1/3}, \end{array}$$

where $d_{r}$ is the diameter of the bottom of the screw, E is Young’s modulus, K is the stiffness value in the specification table, $F_{pzt}$ is the piezoelectric ceramic output force, and C is the dynamic load in the specification table.

The feed system equivalent damping coefficient $C_{T}$ is:

(9) $$\begin{array}{c} C_{T}=\frac{C_{0}+C_{1}+C_{2}+C_{S}+C_{F}}{K_{1}}. \end{array}$$

where $C_{2}$ is the viscous damping coefficient of the nut assembly, $C_{S}$ is the viscous damping coefficient at the support bearing, and $C_{F}$ is the viscous damping coefficient at the fixed bearing.

Relationship between drive torque and armature current of a servomotor:

(10) $$\begin{array}{c} \gamma =\frac{l}{x}. \end{array}$$

where γ is the inclination angle of the swash plate, because γ is very small, so sinγ≈γ, l is the distance between the point of force applied by the ball screw to the swash plate and the articulation point of the swash plate. From Equation (8), the swash plate inclination position control problem can be converted into a servomotor-driven ball screw nut position control problem.

### 3.3. Servo Motor Model

According to Kirchhoff’s law, the armature voltage equation of a servomotor is:

(11) $$\begin{array}{c} V=L\frac{di}{dt}+iR+V_{e}. \end{array}$$

where V is the motor armature input electromotive force, L is the motor armature circuit inductance, i is the motor armature current, R is the motor armature circuit resistance, and $V_{e}$ is the armature counter electromotive force.

Relationship between armature back-emf and output angular velocity of the motor:

(12) $$\begin{array}{c} V_{e}=C_{e}\dot{\theta_{1}}. \end{array}$$

where $C_{e}$ is the motor back-emf coefficient.

The relationship between the driving torque of the servo motor and the current of the armature:

(13) $$\begin{array}{c} T_{m}=K_{2}i. \end{array}$$

where $K_{2}$ is the torque conversion factor.

### 3.4. LuGre Friction Model

LuGre friction model is a widespread method for friction modelling of electromechanical systems. It is capable of describing static, dynamic, and velocity-dependent friction characteristics [25,26]. The friction generated between the nut assembly and the guideway is meticulously described using the LuGre model, a widely recognized and robust approach to capturing the complex dynamics of friction interactions.

The LuGre friction model can be expressed as follows:

(14) $$\begin{array}{c} F_{f}=\sigma_{0}z+\sigma_{1}\dot{z}+\sigma_{2}\dot{x}. \end{array}$$

where $\sigma_{0}$ is the stiffness coefficient, $\sigma_{1}$ is the viscous damping coefficient, $\sigma_{2}$ is the velocity-dependent viscous damping coefficient, and z is the internal state variable. The rate of change of internal state variables $\dot{z}$ can be expressed as:

(15) $$\begin{array}{c} \dot{z}=\dot{x}-\frac{\left|\dot{x}\right|}{G(\dot{x})}z. \end{array}$$

where $\dot{x}$ denotes the relative tangential velocity of the two surfaces in contact and $G(\dot{x})$ is the Stribeck effect function, which can usually be expressed as:

(16) $$\begin{array}{c} G(\dot{x})=F_{c}+{\left(F_{s}-F_{c}\right)e}^{-{\left(\frac{\dot{x}}{v_{s}}\right)}^{2}}. \end{array}$$

where $F_{c}$ is the dynamic friction, $F_{s}$ is the maximum value of static friction, and $v_{s}$ is the Stribeck velocity parameter used to determine the speed at which G tends to $F_{c}$.

## 4. Mathematical Modeling of Piezoelectric Actuator

### 4.1. Enhanced Bouc–Wen Model

Given that the classical Bouc–Wen model is an approximate centrosymmetric model, it typically results in hysteresis curves that do not accurately represent the asymmetry observed in real systems. In their study of magnetorheological (MR) dampers, Wang et al. [27] similarly encountered the limitations of the classical Bouc–Wen model in describing the force-displacement hysteresis curves of MR dampers. Specifically, the pronounced necking phenomenon observed when current flows through the dampers cannot be accurately characterized by the traditional model. The inability of the Bouc–Wen standard model to adequately capture the actual physical initial state of the system highlights the necessity for improvements [28].

In an improvement of the classical Bouc–Wen model, the model is optimized by introducing nonlinear components to more accurately capture asymmetric hysteresis characteristics in real systems. Specifically, by replacing the coefficients of the linear component with a third-order polynomial form, the model is able to characterize asymmetry of the hysteresis curve more effectively. This improvement not only improves the adaptability of the model but also enhances its accuracy and reliability in simulating the hysteresis behavior of complex systems. The improved Bouc–Wen model is:

(17) $$\left\{\begin{array}{l} y=k_{1}u^{3}+k_{2}u^{2}+d_{p}u-h\\ \dot{h}=\alpha \dot{u}-\beta \left|\dot{u}\right|h-\lambda \dot{u}\left|h\right|, \end{array}\right.\;$$

where u is the input voltage, y is the output, $d_{p}$ is the piezoelectric factor, h represents the hysteresis nonlinear term, $\dot{h}$ is the differentiation of h with respect to t, and $k_{1}$ and $k_{2}$ are the nonlinear term coefficients. α controls the hysteresis loop amplitude, and β and λ control the shape of the hysteresis loop.

### 4.2. Parameter Identification Methods

Particle swarm optimization (PSO) is an optimization algorithm rooted in swarm intelligence principles, stemming from the social behaviors observed in flocks of birds or schools of fish. Within the PSO framework, each candidate solution to the problem is modeled as a “particle” navigating through a multidimensional solution space. Each particle not only has a position but also has velocity, guiding its movement through the search space [29,30]. The specific algorithm is as follows:

(18) $$v_{i+1}=w\times v_{i}+c_{1}\times \mathrm{rand}\left(\right)\times \left({\mathrm{pb}}_{i}-x_{i}\right)+c_{2}\times \mathrm{rand}\left(\right)\times \left(\mathrm{gb}-x_{i}\right),\;$$

(19) $$\begin{array}{c} x_{i+1}=x_{i}+v_{i+1}. \end{array}$$

where, $v_{i}$ is the moving speed of the *i*th particle, $x_{i}$ is the current position of the ith particle. ${\mathrm{pb}}_{i}$ is the optimal solution of the ith particle, gb is the optimal solution of the whole population. Equation (18) consists of three parts. The first part indicates that the particles are affected by the last velocity and inertia, the second part indicates that the particles are affected by their own experience, and the third part reflects the social experience sharing of the particle population, which determines the next movement of the particles.

In the optimization search process, the basic PSO algorithm is prone to converging prematurely on local optima. When dealing with multi-parameter optimization problems, this tendency can lead to inefficiencies in the algorithm and reduced accuracy in the search results. A primary reason for these problems is that inertia weights and learning factors in the basic PSO are typically set as constants. This static configuration lacks dynamic adjustment capability, meaning that these parameters cannot be adjusted in real time as iterations progress [31]. Consequently, the algorithm may become trapped in suboptimal solutions.

To refine the adjustment mechanism of the inertia weights, the *tanh* function was utilized, which allows for a transition from a linear to a nonlinear decrease pattern. This approach allows for more dynamic and adaptive weight adjustments during the optimization process. Dynamic inertia weight adjustment is implemented to enhance exploration capabilities in the initial stages of the algorithm and to strengthen exploitation capabilities in the later stages. The dynamic inertia weights nonlinear change is calculated according to the following equation:

(20) $$\begin{array}{c} w=w_{max}-\left(w_{max}-w_{min}\right)\tanh\left(ai\right). \end{array}$$

where a is the adjustment factor and i is the number of iterations.

During the initial phases of the search, when the algorithm needs more exploration, $c_{1}$ takes a larger value while $c_{2}$ takes a smaller value, which makes the particles more inclined to move towards their personal best position and helps the global search. Upon increasing the number of iterations, the algorithm gradually enters the late stage of searching, when $c_{1}$ decreases and $c_{2}$ increases, which prompts the particles to move more towards the global optimal position and strengthens the ability to search locally, thus converging to the global optimal solution more quickly. The dynamic learning factor is calculated according to the following formula:

(21) $$\begin{array}{c} c_{1}\left(i\right)=c_{1,\;\max\;}-\frac{\left(c_{1,\;\max\;}-c_{1,\;\min\;}\right)\cdot i}{N},\\ c_{2}\left(i\right)=c_{2,\;\min\;}+\frac{\left(c_{2,\;\max\;}-c_{2,\;\min\;}\right)\cdot i}{N}. \end{array}\;$$

where N is the maximum number of iterations.

### 4.3. Model Verification

**Figure 2:**
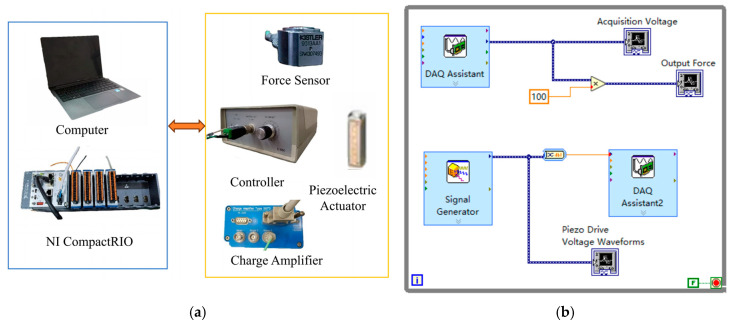
(**a**) Schematic diagram of experimental equipment. (**b**) LabVIEW experimental data acquisition program.

As illustrated in Figure 2a, the experimental setup is designed to enable the identification and calibrate the Bouc–Wen model, providing a structured approach for parameter estimation. The specific parameters of the piezoelectric ceramics used are detailed in Table 1. The data acquisition program is written in LabVIEW using the DAQ assistant, as shown in Figure 2b. LabVIEW is a graphical programming environment widely used in the fields of test and measurement, data acquisition, and instrumentation control. The DAQ assistant is a convenient data acquisition tool in LabVIEW that simplifies interaction with data acquisition hardware, allowing users to quickly set up and run data acquisition tasks. The signal input is generated by NI Compact RIO’s AO module, and a sine voltage signal with a frequency of 1 Hz, amplitude of 30 V, and deviation of 30 V is used as input to the piezoelectric ceramic by the controller. The output force of the piezoelectric ceramic was measured by the pressure transducer in conjunction with the charge amplifier. Finally, these test data are used in parameter identification in the enhanced Bouc–Wen model described above.

**Figure 3:**
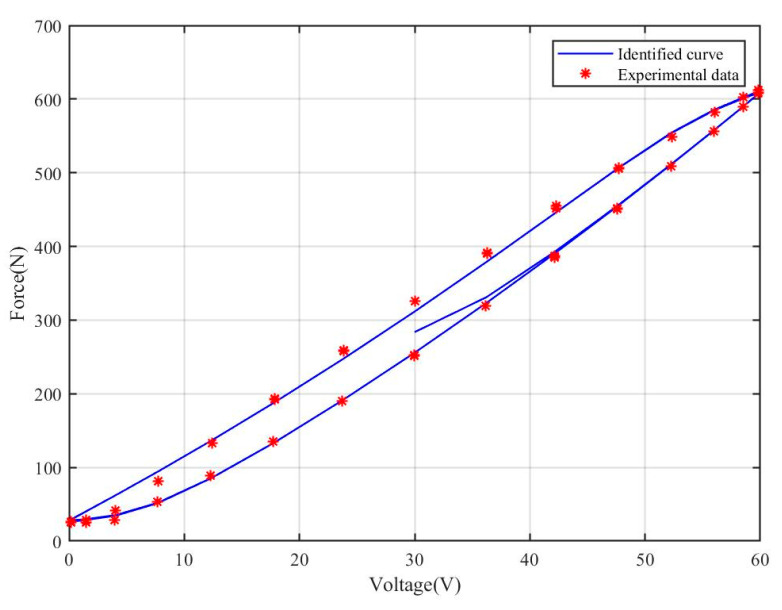
Hysteresis curves of piezoelectric ceramics.

**Figure 4:**
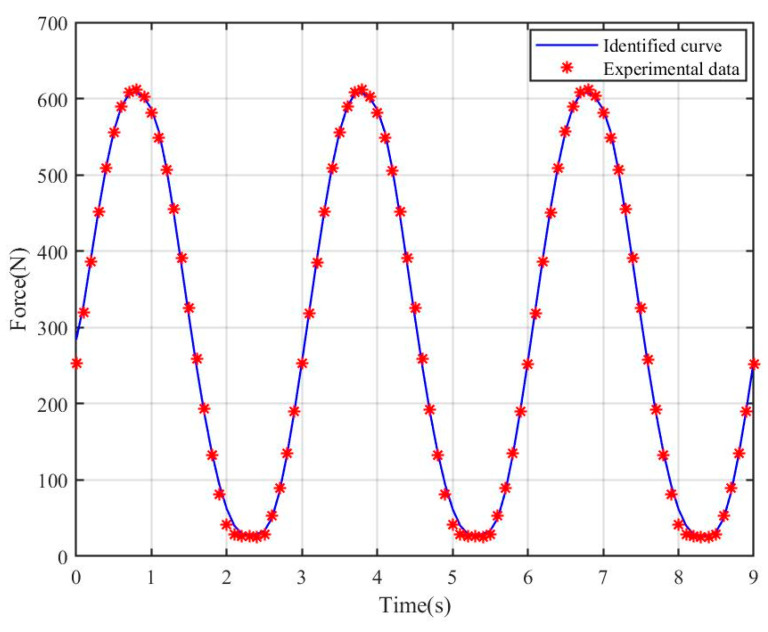
Identified curves for improved asymmetric Bouc–Wen model.

According to the results demonstrated in Figure 3 and Figure 4, after a series of parameter optimizations and adjustments, the improved Bouc–Wen model exhibits a significantly improved fit effect. The model not only captures the dynamic characteristics of the system more accurately, but also its prediction performance is improved, and the discrepancy with the actual measured data is significantly reduced.

To enhance the model’s ability to accurately describe real-world physical processes, we introduced additional nonlinear terms within the theoretical framework. This enhancement not only boosts the prediction accuracy of the model for nonlinear systems, but also significantly increases its flexibility and adaptability under different operating conditions. Particularly in simulating complex hysteresis phenomena, the improved Bouc–Wen model demonstrates a superior ability to closely replicate real-world physical processes, providing a more robust basis for engineering design and analysis.

**Figure 5:**
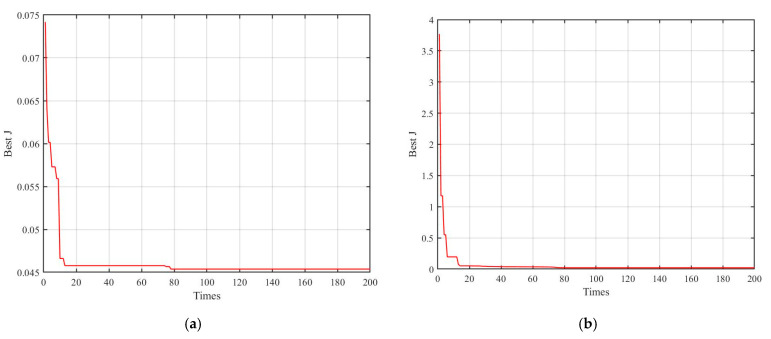
Comparison of fitness value of identification results: (**a**) fitness value of 0.0454. (**b**) fitness value of 0.0215.

As illustrated in Figure 5, a comparison of fitness curves for the PSO algorithm before and after improvements reveals a significant improvement in optimization performance following the introduction of dynamic inertia weights and learning factors. The refined PSO algorithm not only achieves superior optimization accuracy, with a fitness value of 0.0215, but also exhibits significantly faster convergence rate throughout the iterative process. The implementation of dynamically adjusted inertia weights and learning factors facilitates an optimal balance between exploration and exploitation, thus enhancing the robustness and efficiency of the algorithm in locating the global optima. This approach ensures that particles can navigate the search space more efficiently, leading to improved solution quality and reliability. The results of parameter identification for the improved Bouc–Wen model are summarized in Table 2, highlighting the effectiveness of these enhancements.

**Figure 6:**
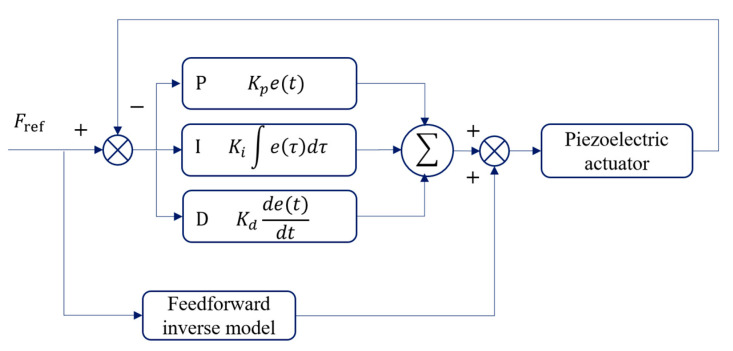
Feedforward–feedback compensation method based on hysteresis inverse model.

Following the successful identification of Bouc–Wen model parameters using the improved PSO algorithm, we further implemented a composite control strategy combining feedforward inverse modeling with PID feedback control to enhance the system’s control accuracy and response speed. The control block diagram depicted in Figure 6 illustrates the architecture of this integrated control system.

In this framework, the feedforward inverse model serves as a compensation link, generating recompensated signals based on the nonlinear characteristics predicted by the Bouc–Wen model. This proactive approach effectively counteracts the inherent nonlinearities of the system, ensuring that the output remains consistent with the expected trajectory. Meanwhile, the PID controller, an integral part of the feedback loop, addresses any residual errors not fully compensated by the feedforward mechanism. This dual-layered control strategy ensures robust performance, maintaining high control fidelity even when confronted with complex dynamic behaviors.

## 5. Results and Discussion

**Figure 7:**
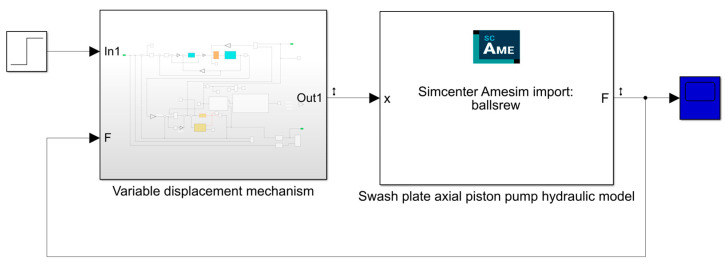
Swash plate axial piston pump model.

**Figure 8:**
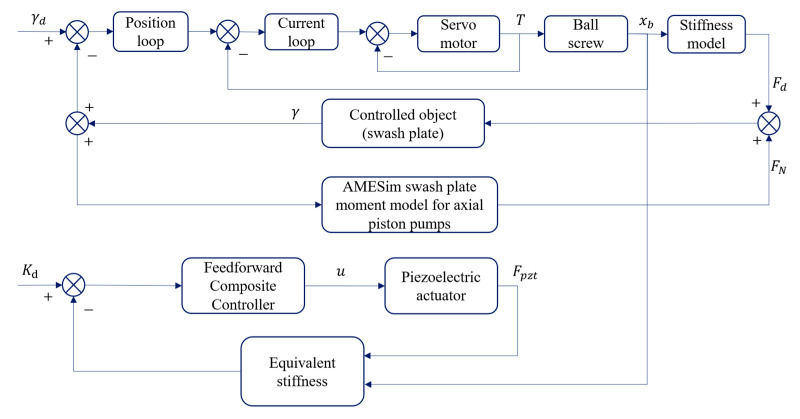
System overall structure control block diagram.

To thoroughly analyze the improvement potential of the electronically controlled variable displacement mechanism compared to the traditional valve-controlled variable displacement mechanism in terms of response speed, control accuracy, and overall dynamic performance, we developed a ball screw simulation model actuated by a piezoelectric actuator in the Simulink environment. This model is based on the previously established mathematical framework. In addition, a hydraulic model of axial piston pump containing modules such as plunger vice, distributor disk vice and swash plate torque was built on the AMESim platform to simulate in detail the fluid dynamics inside the pump chamber, the influence of the swash plate position on displacement and the interaction with the external hydraulic circuit. This study utilizes the co-simulation technology of AMESim and Simulink and realizes the efficient integration of the two through the co-simulation framework shown in Figure 7. We built a comprehensive simulation model of the entire axial piston pump system, incorporating an electronically controlled variable displacement mechanism. This model integrates the Simulink environment with AMESim through an interactive interface, using AMESim’s interface module to facilitate seamless coupling between the two platforms. The force applied to the swash plate is transferred through the interface module to the variable displacement mechanism model built in Simulink, ensuring the transfer and response of the force throughout the whole system. Figure 8 shows the control of the integrated system in a block diagram, clearly delineating the interconnections and data exchange paths among its components. The primary parameters of the joint simulation model, detailed in Table 3, serve as the basis for ensuring the accuracy and reliability of simulation results. These parameters are critical for validating the model’s performance and providing a robust basis for analyzing the system’s dynamic behavior, response speed, and control accuracy.

Maximum swash plate inclination angle γ $\mathrm{Ball}\;\mathrm{screw}\;\mathrm{guide}\;P_{h}$

**Figure 9:**
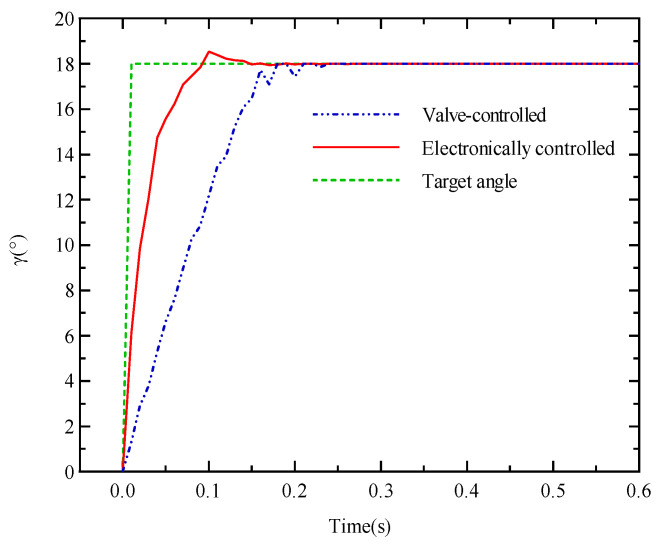
Step comparison curves of swash plate angle for different variable control methods.

Figure 9 presents a comparison of the response characteristics of the electronically controlled variable displacement mechanism and the traditional valve-controlled variable displacement mechanism under target-angle step command conditions. It is worth noting that although both of them adopt closed loop control strategies, the electronically controlled variable displacement mechanism, by virtue of its unique mechanical structure design, goes directly through the servomotor-driven ball screw to regulate the position of the swash plate. This mechanism avoids the complex fluid dynamics process in the traditional hydraulic valve control and greatly shortens the signal transmission path, which significantly reduces the control hysteresis and accelerates the speed of regulation. Excellent dynamic performance is demonstrated. The electronically controlled variable displacement mechanism rapidly approaches the target angle compared to the valve-controlled electronically controlled variable displacement mechanism, with a response time of about 0.06 s. This clearly demonstrates the significant advantages of electronic control technology in improving the dynamic response speed of the system, reflecting the potential of electronic control technology in the design of hydraulic systems. This finding not only verifies the theoretical expectation of electronically controlled design in reducing control hysteresis and speeding up the regulatory process but also provides strong empirical support for the subsequent design and realization of high-performance control systems.

**Figure 10:**
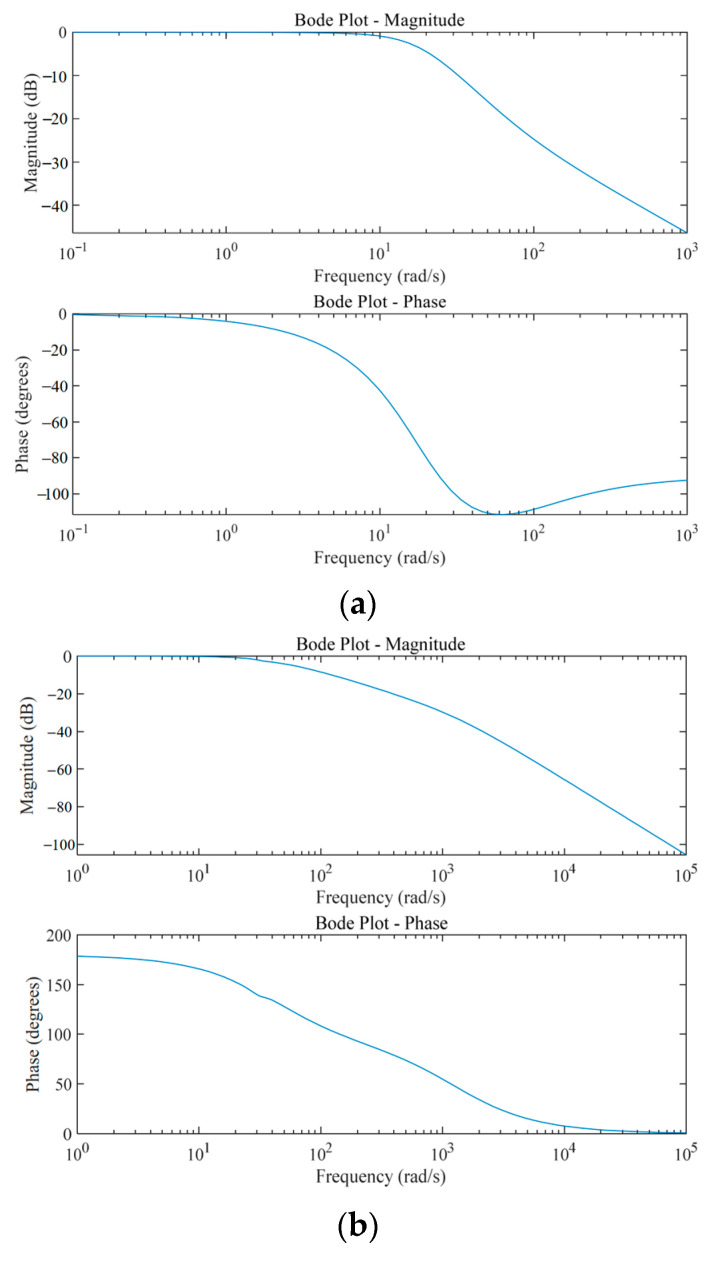
Frequency response characteristic curve: (**a**) valve-controlled variable displacement mechanism; (**b**) electrically controlled variable displacement mechanism.

To comprehensively evaluate and compare the dynamic responses of the electronically controlled and valve-controlled variable displacement mechanisms, a frequency domain analysis was conducted. Converting the signals in the time domain to the frequency domain allows us to visualize the gain and phase changes at different frequency components and helps to compare the dynamic performance of the two control methods. The resulting frequency response characteristic curves are presented in Figure 10.

Based on the results shown in Figure 10, we delve into the significant differences in the dynamic response characteristics between the valve-controlled and electronically controlled variable displacement mechanisms. The cutoff frequency of the valve-controlled variable displacement mechanism shown in Figure 10a is 2.65 Hz at −3 dB, while the cutoff frequency of the electronically controlled variable displacement mechanism shown in Figure 10b is 5.9 Hz. This not only indicates that the electronically controlled variable displacement mechanism has a wider frequency response range but also is able to maintain an effective control accuracy and dynamic adjustment capability in a wider frequency spectrum. The phase Bode plot further reveals the stability advantage of the electronically controlled variable displacement mechanism, which has a larger phase margin and helps to improve the system stability. In contrast, the stability of the valve-controlled variable displacement mechanism may be limited in the high frequency band. Consequently, the electronically controlled system exhibits enhanced adaptability to rapidly changing operating conditions.

**Figure 11:**
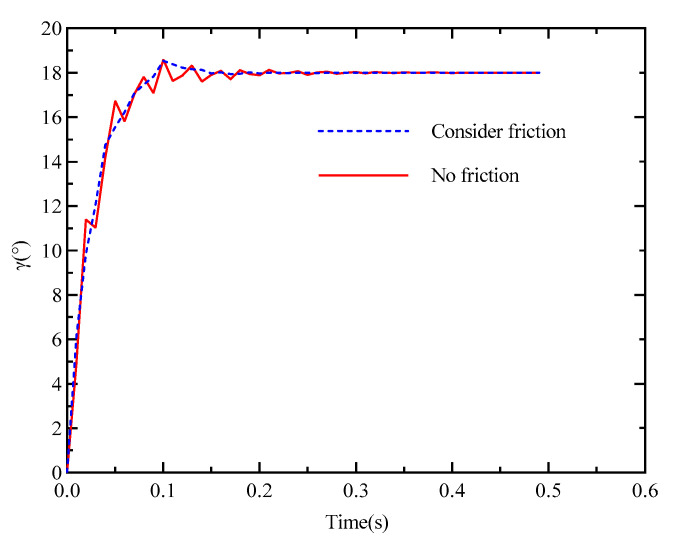
Comparison curves of step response considering friction and without friction.

Figure 11 shows comparative curves of the step response for the electronically con-trolled variable displacement mechanism. The results vividly demonstrate the significant impact of friction on the response characteristics of the system. When friction is taken into account, the system exhibits a smoother response with notably reduced oscillations. Conversely, when friction is ignored, the system’s response becomes more erratic, with increased oscillations. This comparison highlights the critical role of the friction coefficient in achieving stable and precise control performance.

This is due to the fact that the presence of friction in the system acts as a damping effect, reducing oscillations by absorbing the excess energy of the system. The coefficient of friction acts as a “shock absorber” in this case, helping to improve the stability and response quality of the system. However, it is essential to mention that while the friction coefficient plays a beneficial role in reducing oscillations, the complex and nonlinear nature of friction—in particular, its variability with temperature and velocity—can introduce additional challenges in the design and maintenance of control systems for practical applications. In future research, we will more comprehensively consider the impact of friction factors on the control accuracy and stability of the system, aiming to further enhance its overall performance.

**Figure 12:**
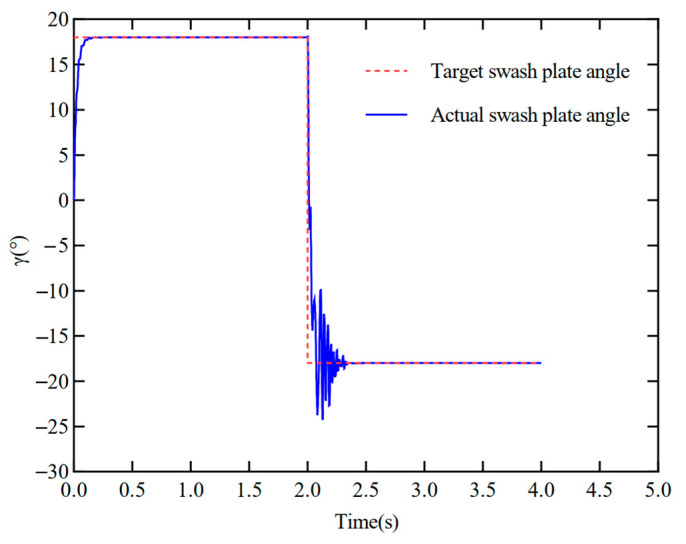
Swash plate positive and negative full-displacement-switching step-tracking curve.

Figure 12 reveals the dynamic response characteristics of the control system during the switching process from positive to negative displacement by the electronically con-trolled variable displacement mechanism. The outcomes indicate that the swash plate inclination angle can be better realized under the regulation of the electronically controlled variable displacement mechanism to switch from positive to negative full displacement, which verifies the accuracy of the model. However, in the switching of displacement, the problems of overshooting and oscillation are generated. Specifically, the maximum overshoot of the system without stiffness compensation reaches 31.8%, which not only affects the response speed of the system but also reduces the accuracy of position switching. Preliminary analysis may be due to the inertia of the swash plate and the drive mechanism causing overshoot in the switching process. Additionally, the hydraulic system contains a variety of nonlinear factors—such as saturation, dead zone, etc.—that may be more significant in the displacement switching process, especially regarding positive displacement switching to negative displacement performance.

**Figure 13:**
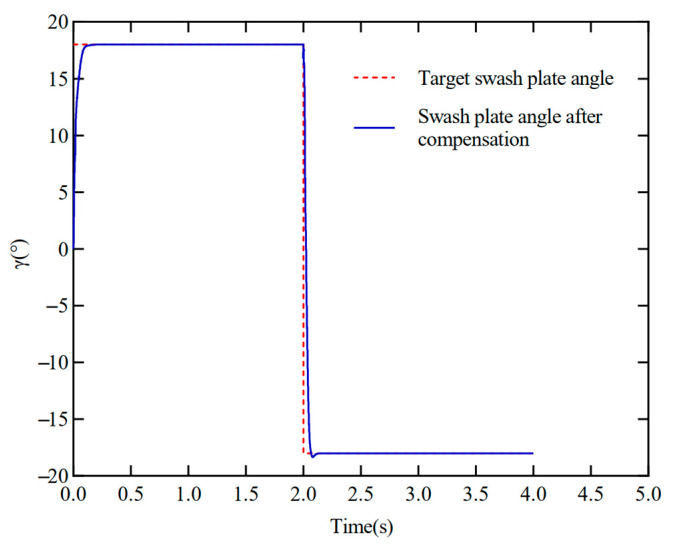
Swash plate camber switching curve with variable stiffness adjustment.

Aiming at the problem of oscillation or overshoot during the switching process of the swash plate, an active adjustment method based on piezoelectric ceramic is proposed in this paper. By precisely controlling the excitation voltage of the piezoelectric element, the local stiffness distribution of the ball screw can be changed in real-time operation. This dynamic tuning function enables the system to immediately adjust its intrinsic frequency and damping characteristics during the swash plate switching period, thus suppressing unwanted vibrations and improving overall dynamics. The swash plate position switching curve under the variable stiffness adjustment of piezoelectric ceramics is shown in Figure 13. The adjusted system exhibits significantly reduced oscillation and overshoot during swash plate position switching. The maximum system overshoot was significantly reduced to 1.9%. In addition, the tuned system exhibits faster settling time. This demonstrates that dynamically adjusting the total stiffness of the ball screw, the system can accomplish the position switching more smoothly and accurately, and the piezoelectric element is able to adjust its output in real time according to the system’s demand, thus changing the stiffness characteristics of the ball screw. This dynamic adjustment mechanism effectively suppresses the nonlinear effect and reduces the instability caused by the stiffness change, making the system more stable and controllable during the switching process.

## 6. Conclusions

In order to overcome the limitations in the existing design of the traditional valve-controlled axial piston pump, this paper develops an electronically controlled variable displacement control mechanism with stiffness compensation based on piezoelectric actuators. The vibration of the swash plate is closely related to its stiffness, and its vibration characteristics can be improved by changing its stiffness. Therefore, a piezoelectric element is introduced into the ball screw mechanically connected to the swash plate to compensate for the stiffness of the entire system, thus improving the vibration characteristics of the swash plate and enhancing the stability and dynamic performance of the system. Through mathematical modeling and simulation analysis, it is found that the response speed of the electronically controlled variable displacement mechanism is significantly better than that of the traditional valve-controlled mechanism under specific conditions, and the system bandwidth is higher so that it can better adapt to changes in dynamic working conditions and improve the regulation capability of rapidly changing loads. In order to address the issue of poor system stability resulting from the alternating reaction force of the swash plate during the operation of the ball screw, this paper introduces piezoelectric ceramics characterized by their reversibility, high stiffness, and high frequency response into the nut assembly to achieve stiffness compensation control. Based on this approach, we established a stiffness model for the double nut ball screw and explored the relationship between piezoelectric ceramics and double nut assembly. Furthermore, an asymmetric Bouc–Wen piezoelectric actuator model incorporating nonlinear hysteresis characteristics was developed, and the parameters of this model were identified using an improved PSO algorithm. The results demonstrate that the stiffness compensation system exhibits enhanced stability during both positive and negative full displacement switching of the swash plate. This finding verifies the reliability and efficacy of the proposed electronically controlled variable displacement mechanism and its associated stiffness compensation strategy. In studying the friction factor, it was observed that the reasonable use of friction can significantly improve the stability and control accuracy of the system, while reducing the oscillation of the system response. These findings indicate that the proposed electronically controlled variable displacement mechanism, along with its stiffness compensation method, provides robust support for enhancing the dynamic performance of swash plate axial piston pumps.

## Figures and Tables

**Table 1:** Piezoelectric material properties.

Parameters	Value
Piezo ceramic type	PIC 252
Dimensions A × B × L(mm)	10×10×36
Piezoelectric constant $d_{33}$ (pC/N)	400
Electro-mechanical coupling coefficient $k_{P}$	0.62
Stiffness (N/μm)	100
Blocking force (N)	3800

**Table 2:** Bouc–Wen model parameters.

$k_{1}$	$k_{2}$	$d_{p}$	α	β	λ
−0.0001	0.0423	8.2510	6.3808	0.1381	0.0904

**Table 3:** Swash plate axial piston pump parameters.

Parameters	Value
Plunger diameter D (mm)	22.2
Plunger distribution circle radius r (mm)	49.25
Maximum swash plate inclination angle γ (°)	18
Number of plungers	9
Nominal diameter of ball screw d (mm)	20
Ball screw guide $P_{h}$ (mm)	10

## Data Availability

The data presented in this study are available on request from the corresponding author. Certain data are part of the current study and making them publicly available may have an impact on the integrity of the subsequent study or may even lead to early use or misinterpretation of the data. Therefore, we recommend that the data be made available through the corresponding author to ensure that access can be strictly controlled during data sharing and that the use of the data is properly monitored.
